# Is There a Weekly Pattern for Health Searches on Wikipedia and Is the Pattern Unique to Health Topics?

**DOI:** 10.2196/jmir.5038

**Published:** 2015-12-22

**Authors:** Elia Gabarron, Annie YS Lau, Rolf Wynn

**Affiliations:** ^1^ Norwegian Centre for E-health Research University Hospital of North Norway Tromsø Norway; ^2^ Faculty of Health Sciences Department of Clinical Medicine The Arctic University of Norway Tromsø Norway; ^3^ Centre for Health Informatics Australian Institute of Health Innovation Macquarie University Sydney Australia

**Keywords:** information-seeking behavior, health information–seeking behavior, periodicity, Wikipedia, chlamydia, gonorrhea, HIV, AIDS, influenza, diabetes

## Abstract

**Background:**

Online health information–seeking behaviors have been reported to be more common at the beginning of the workweek. This behavior pattern has been interpreted as a kind of “healthy new start” or “fresh start” due to regrets or attempts to compensate for unhealthy behavior or poor choices made during the weekend. However, the observations regarding the most common health information–seeking day were based only on the analyses of users’ behaviors with websites on health or on online health-related searches. We wanted to confirm if this pattern could be found in searches of Wikipedia on health-related topics and also if this search pattern was unique to health-related topics or if it could represent a more general pattern of online information searching—which could be of relevance even beyond the health sector.

**Objective:**

The aim was to examine the degree to which the search pattern described previously was specific to health-related information seeking or whether similar patterns could be found in other types of information-seeking behavior.

**Methods:**

We extracted the number of searches performed on Wikipedia in the Norwegian language for 911 days for the most common sexually transmitted diseases (chlamydia, gonorrhea, herpes, human immunodeficiency virus [HIV], and acquired immune deficiency syndrome [AIDS]), other health-related topics (influenza, diabetes, and menopause), and 2 nonhealth-related topics (footballer Lionel Messi and pop singer Justin Bieber). The search dates were classified according to the day of the week and ANOVA tests were used to compare the average number of hits per day of the week.

**Results:**

The ANOVA tests showed that the sexually transmitted disease queries had their highest peaks on Tuesdays (*P*<.001) and the fewest searches on Saturdays. The other health topics also showed a weekly pattern, with the highest peaks early in the week and lower numbers on Saturdays (*P*<.001). Footballer Lionel Messi had the highest mean number of hits on Tuesdays and Wednesdays, whereas pop singer Justin Bieber had the most hits on Tuesdays. Both these tracked search queries also showed significantly lower numbers on Saturdays (*P*<.001).

**Conclusions:**

Our study supports prior studies finding an increase in health information searching at the beginning of the workweek. However, we also found a similar pattern for 2 randomly chosen nonhealth-related terms, which may suggest that the search pattern is not unique to health-related searches. The results are potentially relevant beyond the field of health and our preliminary findings need to be further explored in future studies involving a broader range of nonhealth-related searches.

## Introduction

People tend to structure their activities in a weekly (circaseptan) pattern [1], with some days for work and some for rest. The social construct of a 7-day cycle is not new and seems to have its origin around 500 BC [1]. In modern society, many behaviors typically follow a weekly pattern, as do many somatic and psychological symptoms and disorders. For instance, heart attacks, strokes, and migraines tend to be most frequent during the workweek [2-4]. These patterns may be related to differences in physical activity, blood pressure, and stress levels between the workweek and days off from work. Other health activities and aspirational behaviors, such as attending gym, being on time, or quitting smoking, have also been found to be more frequent at the beginning of the week and after temporal landmarks (ie, relocation, job change, birthday, first day of spring). This has been called the “fresh start effect” [5,6].

Sleeping is another central behavior that follows a weekly pattern, typically with a lack of sleep during the workweek (“social jet lag”) and compensatory sleeping on days off [7,8]. Mood and level of aggression also follow various cycles; for instance, suicides are most frequent on Mondays and panic attacks are most frequent on days off from work [9-12].

Sexual risk behaviors, which typically are related to drugs or alcohol consumption, have been reported to be more frequent during the weekends [13,14], whereas online health information-seeking behaviors seem to be more common at the beginning of the workweek [14-16]. Interestingly, the increased rates in online health information-seeking behavior at the beginning of the week have been interpreted as regrets or attempts to compensate for unhealthy behavior or poor choices made during the weekend [14-16] and also as a kind of “healthy new start” [17,18] in agreement with the “fresh start effect” hypotheses [6]. These hypotheses explain the increased information-seeking activity at the beginning of the week as an “aspirational behavior.” This implies that these days (ie, at the beginning of the workweek) would be when people are most motivated to pursue their aspirations or most likely to think about their health [6,17,18].

In this sense, a Spanish website on sexual health found peaks in the number of consultations received by phone and email on Mondays and Tuesdays [15] and a Dutch website on sexual health reported peaks in their visitor rates every Monday [16]. The searches performed on Google seem to follow the same weekly pattern; analyses of the number of US searches including the term “healthy” or “diet” as well as smoking cessation queries showed peaks at the beginning of the week [6,17,18]. The analyses of the number of postings on online smoking cessation support networks also have weekly patterns, with highest activity during the workweek and lower numbers of postings on Saturdays [19].

In these studies, the observations regarding the most common health information-seeking day are based only on the analyses of users’ behaviors with websites on health or on health-related searches on the Google engine. We wanted to examine if the pattern described previously could also be observed in searches on Wikipedia and to what degree the search pattern was specific to health-related information seeking or whether similar patterns could be found in nonhealth-related information-seeking behavior. Therefore, to test this idea, we hypothesized that the information-seeking pattern with peaks in searches at the beginning of the workweek was specific to health-related information seeking. If a similar pattern for nonhealth-related topics was also found, this could mean that a more general pattern of online information searching existed—which could be of relevance even beyond the health sector. The information-seeking behavior shown by people who search Wikipedia can be considered to be representative for online information-seeking behavior patterns in general. Qualitative studies have found that accessibility, perceived trustworthiness, and usability are the most important criteria for online health information seekers [20,21]. Moreover, Wikipedia might represent one of the most frequently used online resources for information seeking and health information seeking in countries with high Internet penetration rates, such as Norway [22]. Wikipedia appears on the first page of Google search results, it is considered a trustable source of information on health [23,24], and the information on Wikipedia is even used by health professionals and researchers [25].

## Methods

Wikipedia has become one of the main sources of information on the Internet [26,27]. Its excellent Web positioning / search engine optimization makes Wikipedia one of the first hits after searching almost any word on the Internet. Currently, it has more than 35 million articles written in 288 different languages and the Norwegian edition, with 413,459 articles, represents the 19th largest language edition [27]. By the end of September 2015, the Norwegian Wikipedia edition had 58,706 page views per hour. Hypothetically, if each of these searches were performed by different individuals, this could imply that 28% of Norwegians accessed Wikipedia daily [28]. Although this figure is likely too high, the point stands that Wikipedia has become a central source of online information for Norwegians.

To examine the search patterns related to health-related topics and nonhealth-related topics, we tracked the number of searches performed in the Norwegian language on Wikipedia from January 1, 2013 to June 30, 2015.

The traffic statistics was extracted from 10 Wikipedia articles. Because there have been several prior publications on the weekly pattern of information seeking about sexually transmitted diseases (STDs), we chose to examine searches for 5 of the most common STDs (chlamydia, gonorrhea, herpes, human immunodeficiency virus [HIV], and acquired immune deficiency syndrome [AIDS]). To get a broader picture of the search pattern for diseases, we also chose to include a common seasonal infectious disease (influenza), which could present a more seasonal pattern. We also chose to include a common noninfectious disease (diabetes) [29] as well as a natural bodily process (menopause) to further broaden the scope of the searches. To test our hypothesis that the search pattern with peaks at the beginning of the workweek would be specific to health-related information searching, we included 2 randomly chosen celebrities in sports and music: 3-time FIFA Ballon d’Or winner Lionel Messi and one of the current top-selling pop stars, Justin Bieber. Thus, the articles we tracked in this study were chlamydia, gonorrhea, herpes, HIV, AIDS, influenza, diabetes, menopause, Justin Bieber, and Lionel Messi.

The daily rates of Wikipedia article hits were extracted from the Wikipedia article traffic statistics website [30]. This website counts Wikipedia page views per day and classifies the views by the article titles [30]. The dates were classified according to the day of the week. Public holidays in Norway and days after public holidays were also identified.

Descriptive statistics were used to summarize the absolute numbers and frequencies of hits per day. ANOVA tests were used to compare the means of hits per day of the week. The Mann-Whitney *U* test was used to compare the means of hits during public holidays and the first day after the public holidays. Data were analyzed with SPSS version 22.

## Results

**Figure 1:**
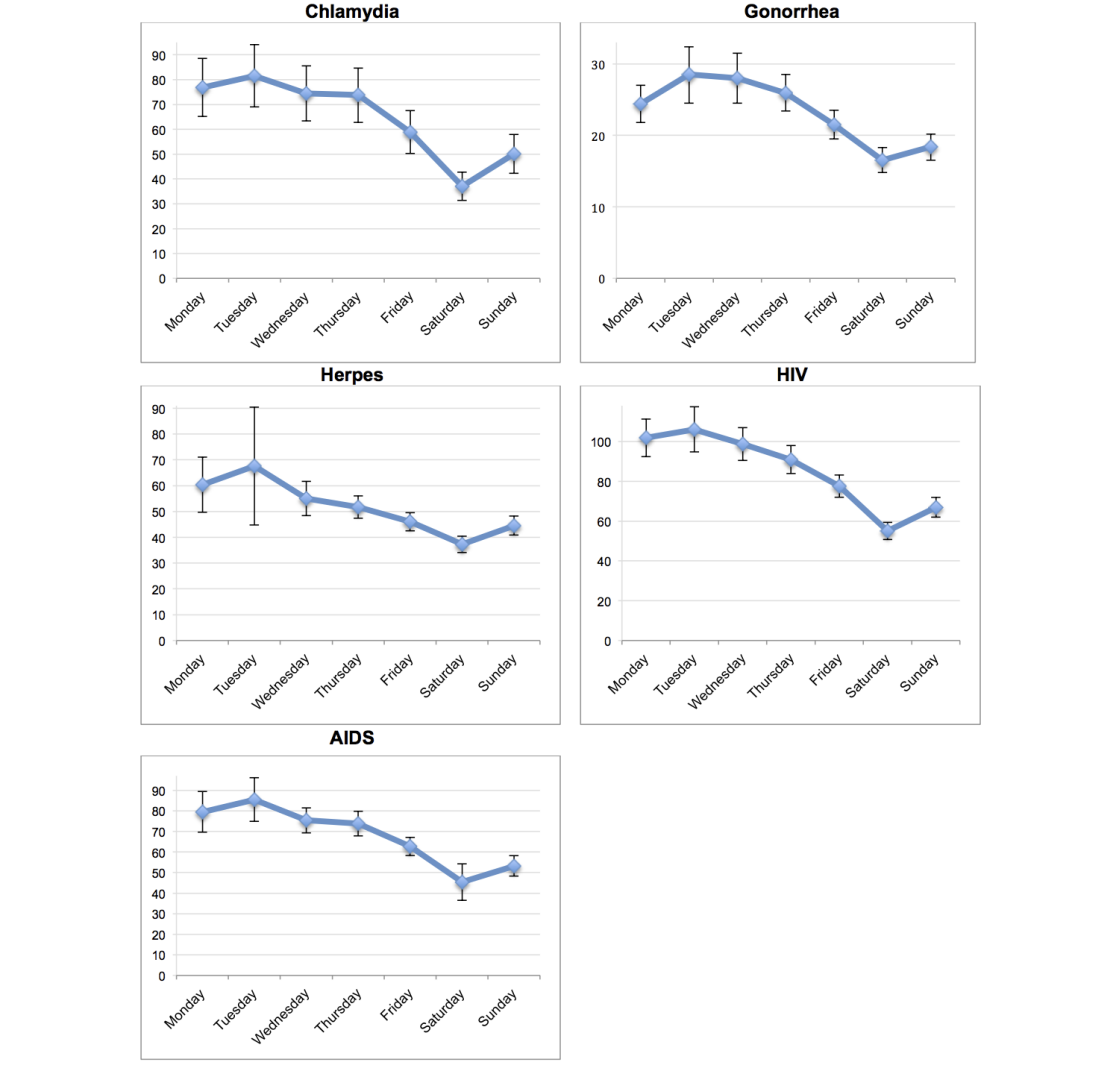
Weekly mean online information searches on sexual diseases (January 1, 2013-June 30, 2015). Error bars indicate 95% CI. All ANOVA tests P<.001.

**Figure 2:**
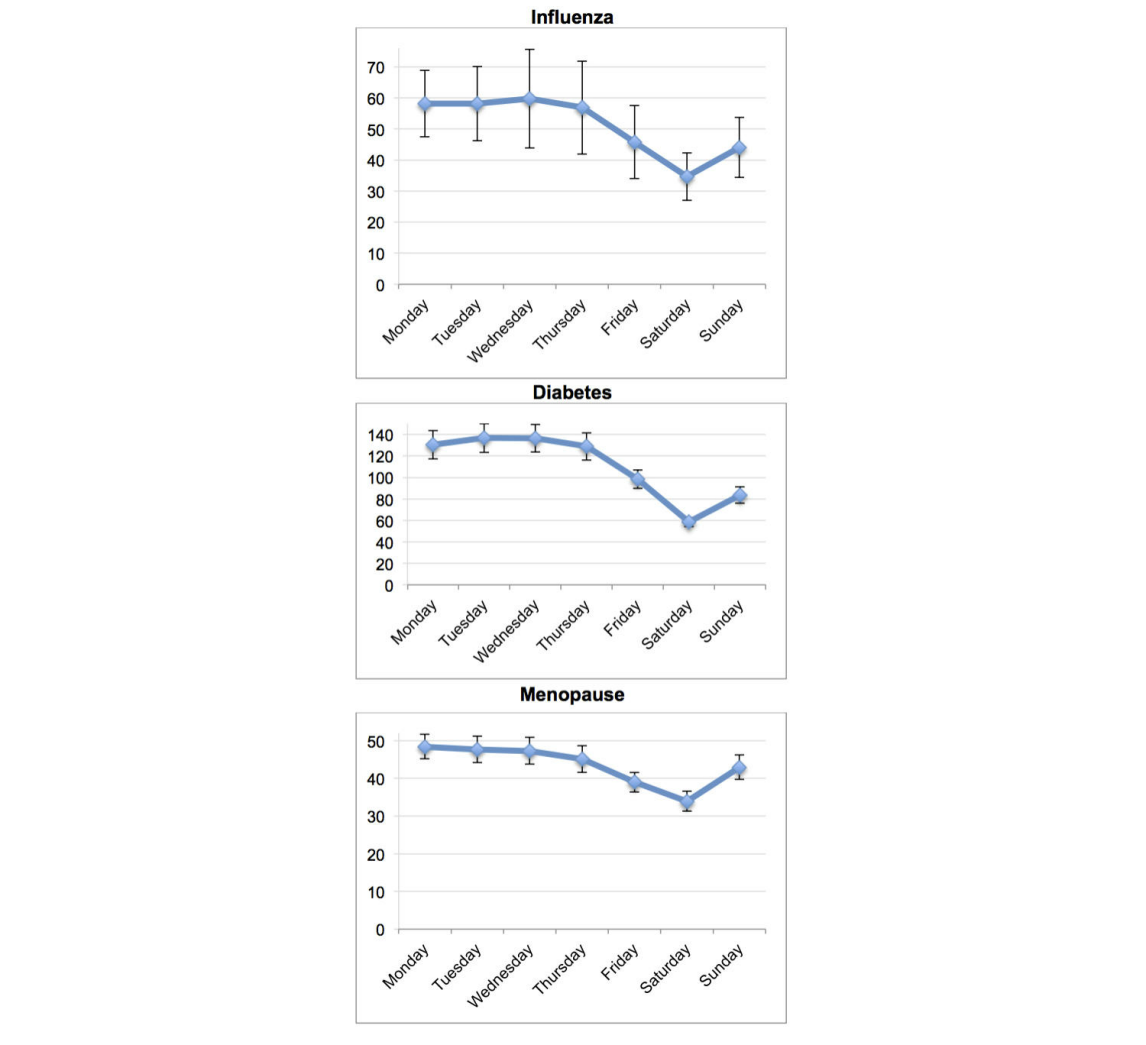
Weekly mean online information searches on other health topics (January 1, 2013-June 30, 2015). Error bars indicate 95% CI. All ANOVA tests P<.001.

**Figure 3:**
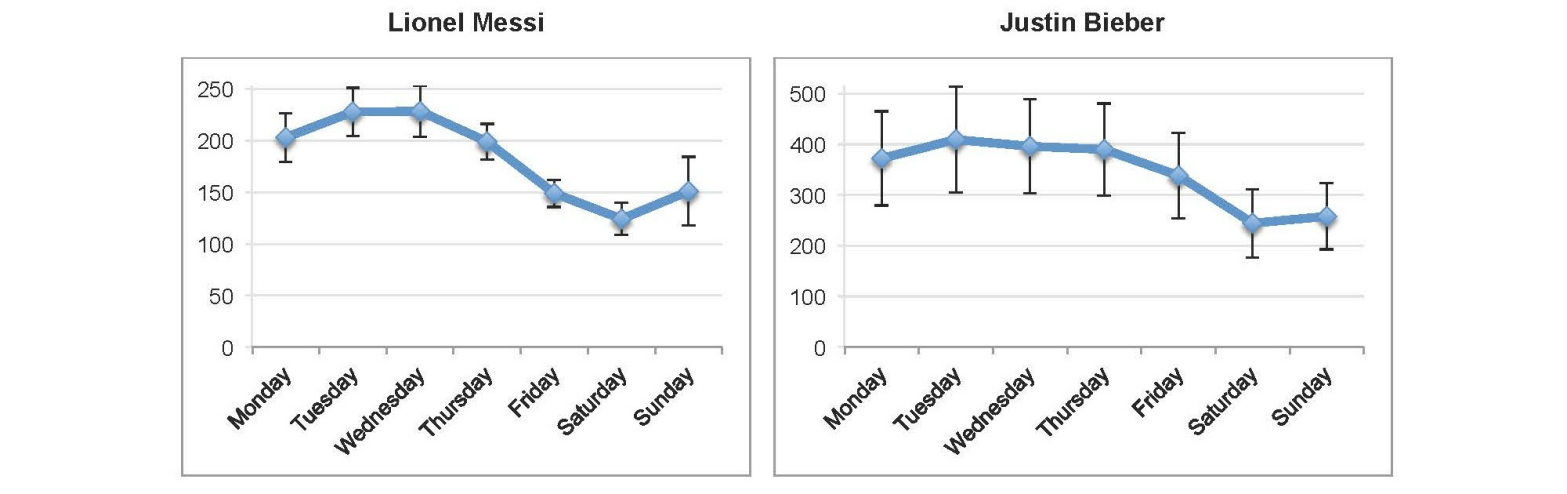
Weekly mean online information searches on nonhealth-related topics (January 1, 2013-June 30, 2015). Error bars indicate 95% CI. All ANOVA tests P<.05.

A total of 10 articles on Wikipedia were tracked for 911 days (from January 1, 2013 to June 30, 2015). All the tracked Wikipedia hits showed a significant weekly pattern with highest peaks early in the week, mostly on Mondays and Tuesdays, and lower numbers on Saturdays. The daily mean queries per search term and their 95% confidence intervals are summarized in Table 1 and Figures 1-3.

**Table 1:** Mean weekly Wikipedia traffic (January 1, 2013-June 30, 2015).

Wikipedia information searches		Day of week, mean (95% CI)							*P* ^a^
		Monday	Tuesday	Wednesday	Thursday	Friday	Saturday	Sunday	
**Sexual diseases**									
	Chlamydia	76.8 (65.2-88.5)	81.5 (69.0-94.0)	74.5 (63.4-85.6)	73.8 (62.8-84.7)	58.9 (50.3-67.5)	37.1 (31.4-42.7)	50.1 (42.3-57.9)	<.001
	Gonorrhea	24.4 (21.8-27.0)	28.5 (24.5-32.4)	28.0 (24.5-31.5)	25.9 (23.4-28.5)	21.5 (19.5-23.5)	16.5 (14.8-18.3)	18.4 (16.5-20.2)	<.001
	Herpes	60.4 (49.7-71.1)	67.6 (44.7-90.5)	55.0 (48.4-61.7)	51.7 (47.4-56.1)	46.0 (42.5-49.5)	37.3 (34.1-40.5)	44.5 (40.8-48.2)	<.001
	HIV	101.9 (92.4-111.3)	106.1 (94.7-117.4)	98.7 (90.5-106.9)	90.9 (83.9-97.9)	77.5 (72.0-83.1)	55.0 (50.7-59.3)	66.9 (62.0-71.9)	<.001
	AIDS	79.5 (69.6-89.5)	85.5 (74.9-96.2)	75.4 (69.3-81.5)	73.8 (67.8-79.8)	62.8 (58.3-67.2)	45.4 (36.5-54.3)	53.2 (48.3-58.2)	<.001
**Other health topics**									
	Influenza	58.2 (47.5-68.9)	58.2 (46.2-70.1)	59.8 (43.9-75.7)	56.9 (41.9-71.8)	45.7 (34.0-57.5)	34.7 (27.0-42.3)	44.0 (34.4-53.7)	<.001
	Diabetes	130.4 (117.2-143.5)	136.8 (123.4-150.1)	136.5 (123.7-149.2)	128.9 (116.2-141.5)	98.4 (89.9-106.9)	58.6 (54.2-63.0)	83.6 (76.0-91.2)	<.001
	Menopause	48.4 (45.2-51.7)	47.7 (44.2-51.2)	47.3 (43.8-50.9)	45.1 (41.6-48.7)	39.0 (36.4-41.6)	33.9 (31.3-36.6)	42.9 (39.7-46.2)	<.001
**Nonhealth-related topics**									
	Lionel Messi	203.1 (179.3-226.5)	227.7 (204.5-250.9)	228.1 (203.4-252.9)	199.1 (181.8-216.3)	148.8 (135.6-161.9)	124.1 (108.4-139.9)	151.0 (117.7-184.3)	<.001
	Justin Bieber	371.9 (279.3-464.6)	408.7 (304.5-512.9)	395.9 (303.1-488.7)	389.8 (299.0-480.6)	337.8 (253.2-422.3)	244.0 (176.8-311.2)	258.1 (192.4-323.8)	.03

^a^ ANOVA.

The ANOVA tests showed that the Wikipedia queries on STDs performed in Norwegian had their highest peaks on Tuesdays (*P*<.001) and Saturday was the day with the fewest searches on STDs. Of the STD searches examined, HIV and AIDS were most frequently searched for. For instance, on Tuesdays, there were a mean 106.1 (95% CI 94.7-117.4) hits on HIV and 85.5 (95% CI 74.9-96.2) hits on AIDS. Comparing the number of hits on Tuesdays and Saturdays, the number of hits was 54.5% lower for searches on chlamydia and 48.2%, 46.9%, 44.8%, and 42.1% lower for HIV, AIDS, herpes, and gonorrhea, respectively.

The other health topics searched on Wikipedia also showed a weekly pattern, with the highest peaks early in the week and lower numbers on Saturdays (*P*<.001). The influenza queries had their highest frequency from Monday to Wednesday, with a peak of 59.8 hits on Wednesdays, on average. The diabetes queries were most frequent on Tuesdays and Wednesdays (peaks of 136.8 hits and 136.5 hits, respectively). The term menopause was most frequently searched on Mondays and Tuesdays (mean peaks of 48.4, 95% CI 45.2-51.7 hits and 47.7, 95% CI 44.2-51.2 hits, respectively). The 3 other health topics tracked showed significantly lower numbers on Saturdays (*P*<.001). The number of queries on Wikipedia decreased 29.9% for searches on menopause, 41.9% for influenza, and 57.2% for diabetes between the days with the highest and lowest numbers of hits. See Table 1 and Figure 2.

Barcelona footballer Lionel Messi had the highest mean number of hits on Tuesdays and Wednesdays (227.7, 95% CI 204.5-250.9 and 228.1, 95% CI 203.4-252.9, respectively), whereas celebrity pop singer Justin Bieber had the most hits on Tuesdays (mean 408.7, 95% CI 304.5-512.9). Both tracked search queries also showed significantly lower numbers on Saturdays, with a decrease of 45.6% for Messi and 40.3% for Bieber. See Table 1 and Figure 3.

A total of 37 public holidays in Norway and 18 days following the public holidays were identified. Mann-Whitney *U* tests showed significant mean differences for online searches performed on public holidays and days after holidays only for the searches on HIV (*P*=.01), AIDS (*P*=.03), diabetes (*P*=.01), and Lionel Messi (*P*=.04). No differences were found in the remaining online searches analyzed. The mean and 95% CI of hits on Wikipedia during public holidays and the days after public holidays are summarized in Table 2.

**Table 2:** Wikipedia traffic on public holidays and days after public holidays.

Wikipedia information searches		Public holidays, mean (95% CI) n=37	Days after public holidays, mean (95% CI) n=18	*P* ^a^
**Sexual diseases**				
	Chlamydia	43.3 (29.3-57.3)	66.5 (31.9-101.1)	.30
	Gonorrhea	17.7 (12.7-22.7)	22.1 (16.0-28.1)	>.99
	Herpes	40.8 (33.5-48.1)	55.5 (37.3-73.7)	.11
	HIV	56.0 (47.8-64.2)	81.1 (63.0-99.2)	.01
	AIDS	41.4 (35.3-47.6)	61.2 (44.3-78.1)	.03
**Other health topics**				
	Influenza	41.4 (15.4-67.3)	81.9 (-17.6-184.5)	.30
	Diabetes	69.1 (58.8-79.3)	114.9 (78.0-151.9)	.01
	Menopause	38.2 (31.8-44.7)	44.5 (34.6-54.4)	.24
**Nonhealth-related topics**				
	Lionel Messi	111.2 (85.1-137.3)	170.0 (118.6-221.4)	.04
	Justin Bieber	229.2 (123.1-335.3)	327.8 (134.3.1-521.4)	.47

^a^ Mann-Whitney *U* test (2-tailed).

## Discussion

Our results show that all online queries we examined followed a circaseptan, or weekly, pattern independent of the nature of the search query (sexual diseases, other health topics, or nonhealth-related topics). This weekly online information-seeking pattern has its higher peaks early in the week, mostly on Mondays and Tuesdays, and its low peaks on Saturdays. To our knowledge, this is the first study suggesting the possibility of a weekly pattern in general online information-seeking behavior relating to topics beyond health.

### A Weekly Online Information-Seeking Pattern

Online information-seeking behavior is an intentional, planned behavior that is performed on the Internet. In the case of online searches related to health, one could speculate that the searches are performed mostly by adults with a specific interest in the topics, maybe because they or a loved one have been diagnosed with a condition or because they think they might have a health-related problem [31-33]. Some previous studies have reported weekly patterns of health information-seeking behavior; these findings have been interpreted by some as reflecting people regretting their unhealthy weekend behaviors [14-16] and also as a kind of “healthy new start” or “fresh start” [6,17,18]. Interestingly, in our results, the nonhealth-related queries also followed the same weekly pattern and this implies that the pattern is not unique to health-related searching and there is a need to further examine if online early week information-seeking behavior could represent a more general information-seeking behavior pattern. Furthermore, it is possible that these information-seeking patterns could apply to various age groups because disease-related searches were more likely to have been performed by adults, whereas most searches on the pop singer Justin Bieber were probably performed by teenagers (ie, his main fan base).

Possibly, the online information-seeking behavior pattern could be understood in light of the “fresh start effect” hypothesis in the sense that the higher number of searches on Wikipedia could result from an increased motivation of people to increase their knowledge by seeking information at the beginning of the week [5,6]. However, in our sample, the most common days to perform the online searches were Mondays and Tuesdays, whereas the “fresh start effect” hypothesis would suggest the highest frequency on Mondays only. If we consider the public holidays as a temporal landmark, our results do not fit the “fresh start effect” hypothesis completely. Although the mean number of hits performed on Wikipedia on days after public holidays is higher than the mean number of hits on public holidays, our results show, in all cases, these numbers are lower than the ones observed at the beginning of the week. This could mean that the start of the week has a more powerful effect than temporal landmarks, such as public holidays. On the other hand, the mean number of online hits on Saturdays was, in all our observations, lower than the number of searches performed on public holidays.

### Do New Technologies Have an Effect on the Weekly Behavior Pattern?

In a world where people have constant access to the Internet, it could be that we tend to structure our online information-seeking activities in a weekly pattern; information-seeking behavior activities occur more frequently early in the week, whereas the least popular days for online information searching are Saturdays. In the same way that there are days for work and days for rest, there might be days when online information seeking is more common and days when seeking information online is less frequent. We have not found publications analyzing the existence of information-seeking behavior patterns in the offline world; therefore, we cannot say if these online patterns are just a reflection of usual offline information-seeking behavior. Maybe the weekly online information-seeking pattern might respond to new behavior routines related to the appearance of the new technologies. And, if so, some other behaviors linked to the use of the new technologies could also follow a temporal pattern.

### Relevance of Online Information for Decision Making, Policy Making, and for Public and Private Institutions

Much of modern life is organized in weekly patterns and these patterns are of importance to behavior and to when many symptoms and illnesses occur. Although we are unsure about the underlying causes driving the weekly online information-seeking pattern, we believe the pattern might be relevant to those who wish to reach a largest possible audience with specific information, for instance. If the pattern we described is generic for other sources of online information, it might be important to consider which day of the week that is chosen to release important information to get maximum online exposure. Thus, if confirmed in future studies, the search pattern information could be relevant for press releases or public campaigns of public health agencies, nongovernmental organizations, charities, online publishers, etc. When dealing with this issue, it should also be taken into consideration that people may also be more likely to search for other types of information (ie, not related to health) on the same days. This implies that it will remain a challenge to make the health-related message or campaign attract attention and stand out from the other information that is available.

Increasingly, online information plays an important part in decision making [34]. Decisions are improved by better access to relevant information and searching for documents on the Web is increasingly an important source of that information [35]. However, although past research has focused on population access and usage of the Internet [34,36,37], there has been little, if any, examination of when general information needs or motivations (in addition to health information-seeking behavior) are likely to arise during the week, whether searches are conducted in temporal patterns, and how they influence the actual decisions that are being made in an everyday context [38]. This information could be important in the development of integrated information retrieval systems that support decision making. In agreement with the “fresh start effect” studies, we believe that the implications of these findings may be important for policy making and for public and private institutions targeting the general public because campaigns or messages could have the greatest impact at the beginning of the week when people seem to be most eagerly searching for information [6]. However, more research, including empirical testing, is needed before stronger conclusions can be made.

### Limitations

Our study tracked information regarding 10 article queries performed on Wikipedia in the Norwegian language. This means our findings should be regarded as preliminary and further research should be undertaken to check if these weekly patterns can be found for online searching behavior in general. This implies examining if the pattern stands for a broader range of search terms and in different languages and cultures. Moreover, because Wikipedia searches are anonymous, we do not know how variables such as age, gender, health status, place of living, employment status, etc, impact the searching pattern. These and other variables may be particularly important when conducting public health campaigns. It is also of interest to see to what extent a similar pattern of information seeking can be found for other sources of information, such as social media. It will be interesting to explore the existence of other temporal behavior patterns in relation to the appearance of new technologies and their potential impact on decision making. Although it would be interesting to test our findings in an actual health campaign, this lies beyond the scope of this study.

### Conclusion

Our study supported prior studies finding an increase in health information searching at the beginning of the workweek. However, we also found a similar pattern for 2 randomly chosen nonhealth-related terms, which may suggest that the search pattern is not unique to health-related searches. The results are potentially relevant beyond the field of health and our preliminary findings need to be further explored in future studies involving a broader range of nonhealth-related searches.

## Abbreviations

AIDS: acquired immune deficiency syndrome
HIV: human immunodeficiency virus
STD: sexually transmitted disease
